# Development and application of emotion recognition technology — a systematic literature review

**DOI:** 10.1186/s40359-024-01581-4

**Published:** 2024-02-24

**Authors:** Runfang Guo, Hongfei Guo, Liwen Wang, Mengmeng Chen, Dong Yang, Bin Li

**Affiliations:** 1 The First Affiliated Hospital of Bengbu Medical University, Bengbu Medical University, 287 Changhuai Road, Bengbu, China; 2 School of Public Health, Bengbu Medical University, Bengbu, China; 3 School of Health Management, Bengbu Medical University, Bengbu, China; 4 School of Humanities, Southeast University, Nanjing, China

**Keywords:** Emotion, Recognition, Monitoring, Patients, Medical treatment

## Abstract

**Background:**

There is a mutual influence between emotions and diseases. Thus, the subject of emotions has gained increasing attention.

**Objective:**

The primary objective of this study was to conduct a comprehensive review of the developments in emotion recognition technology over the past decade. This review aimed to gain insights into the trends and real-world effects of emotion recognition technology by examining its practical applications in different settings, including hospitals and home environments.

**Methods:**

This study followed the Preferred Reporting Items for Systematic Reviews (PRISMA) guidelines and included a search of 4 electronic databases, namely, PubMed, Web of Science, Google Scholar and IEEE Xplore, to identify eligible studies published between 2013 and 2023. The quality of the studies was assessed using the Critical Appraisal Skills Programme (CASP) criteria. The key information from the studies, including the study populations, application scenarios, and technological methods employed, was summarized and analyzed.

**Results:**

In a systematic literature review of the 44 studies that we analyzed the development and impact of emotion recognition technology in the field of medicine from three distinct perspectives: “application scenarios,” “techniques of multiple modalities,” and “clinical applications.” The following three impacts were identified: (i) The advancement of emotion recognition technology has facilitated remote emotion recognition and treatment in hospital and home environments by healthcare professionals. (ii) There has been a shift from traditional subjective emotion assessment methods to multimodal emotion recognition methods that are grounded in objective physiological signals. This technological progress is expected to enhance the accuracy of medical diagnosis. (iii) The evolving relationship between emotions and disease throughout diagnosis, intervention, and treatment processes holds clinical significance for real-time emotion monitoring.

**Conclusion:**

These findings indicate that the integration of emotion recognition technology with intelligent devices has led to the development of application systems and models, which provide technological support for the recognition of and interventions for emotions. However, the continuous recognition of emotional changes in dynamic or complex environments will be a focal point of future research.

**Supplementary Information:**

The online version contains supplementary material available at 10.1186/s40359-024-01581-4.

## Introduction

Emotional expression plays a crucial role in human life and work. The earliest definition of “emotion” appeared in the writings of William James (1884), the founder of American psychology. He believed that emotions are sensations of physical change and that any emotion is inevitably accompanied by physiological changes, such as facial expressions, muscle tension, and visceral activity [1]. Similarly, Danish physiologist Lange (1885) presented a similar viewpoint: emotions are not only physiological states that integrate sensations, thoughts, and behaviors but also psychological responses generated by various external stimuli [2]. As a result, researchers in numerous fields have recognized the importance of accurately identifying emotions. In recent years, research on emotion recognition has been applied predominantly in fields such as psychology, affective computing, and clinical therapy.

According to the World Health Organization (WHO), approximately 280 million people worldwide experience depression, with more than 700,000 people dying from suicide [3]. There are many mood-related disorders, such as bipolar disorder (BD), which is characterized by recurrent episodes of alternating mania and depressive symptoms [4, 5]. The manic and pathological states of BD can also be understood as extreme expressions of basic emotions such as sadness, happiness, and disgust. Emotions may be intentionally or unintentionally suppressed, and many individuals might struggle to differentiate between fear and anxiety and between guilt and shame, making it challenging for them to accurately describe complex emotions. Patients with mood-related disorders experience more severe emotional fluctuations than healthy individuals [6], which can, to some extent, reflect the progression of the disease, the risk of relapse, and impaired functioning [7, 8]. Therefore, the continuous monitoring of emotional instability and other variables that may reflect disease activity (such as symptom duration, severity, and frequency) has clinical significance.

Self-monitoring is ubiquitous in the field of psychiatry research. Humans can describe emotions through text, language, or facial expressions and even reflect internal emotions through physiological signals. Emotional charting tools, such as the National Institute of Mental Health’s Life Chart Method (NIMH-LCM) [9], the Symptom Checklist-90-Revised (SCL-90-R) [10], and the Profile of Mood States (POMS), are often used to manage and monitor emotional changes [11]. Due to the sudden spread of COVID-19 and drastic societal changes, emotions are highly susceptible to external influences and are closely related to behavior during the pandemic [12]. To reduce the transmission rate of the novel coronavirus, various personal protective measures and policies aimed at reducing gatherings may pose challenges in measuring emotions [13]. Therefore, simple methods such as voice information or facial expressions may no longer be suitable for emotion monitoring in psychiatry, and perhaps social media could serve as an important source of data [14, 15]. Combining emotional data with mobile phone movement data and linking policies with human behavior can reveal the immense potential of multimodal data in emotion detection [16].

Currently, several intelligent monitoring tools can provide standardized responses to language or behavior and help individuals understand the emotions underlying specific actions [17]. Advancements in wearable devices, mobile terminals, and the Internet of Things (IoT) have provided more efficient multidimensional applications for intelligent emotional monitoring. These methods, which are based on ecological momentary assessment techniques, play an important role in reminding patients to perform self-monitoring [18]. The integration of momentary assessment and sensor data holds significant potential for clinical research and treatment. Sandstrom (2016), in the context of momentary depression and anxiety assessments, combined behavioral data from GPS, accelerometers, and anonymous call records to reveal clinically relevant psychological and behavioral patterns [19]. Effectively constructing an emotion classification model using neurophysiological, facial feature, and behavioral data recorded from portable devices, along with machine learning methods, showcases a novel research area.

This study reviews relevant literature from the past decade to delineate current trends and hotspots in emotion recognition technology, elaborating on its practical applications for patients with mental/physical disorders in both hospital and home environments. Emotional monitoring during patients’ diagnosis, intervention, and treatment has been demonstrated to have a certain effect on reducing morbidity and mortality and improving quality of life. Our goal was to assess the importance and practical application of emotion recognition technology in the treatment of patients with psychological/physical illnesses in the development of psychosomatic medicine. The remaining structure is as follows: Sect. 2 provides a detailed account of the process of collecting and selecting articles for this review. Section 3 provides an overview of emotion recognition methods applied in hospitals and home environments, along with an analysis of their development. Section 4 discusses the contributions of emotion recognition technology to patient treatment and healthcare, highlights the positive and negative impacts, and suggests potential future directions for this research. Finally, Sect. 5 offers a summary of the paper.

## Material collection and research methods

### Retrieval strategy

A literature review was conducted according to the Preferred Reporting Items for Systematic Reviews and Meta-Analyses (PRISMA) guidelines [20]. MeSH terms in Medline were searched. Three categories of keywords were preliminarily identified based on the research question, namely, emotion, recognition, and patients. Emotional-related MeSH terms such as psychology and mental; recognition-related MeSH terms such as express and survey; and patient-related MeSH terms such as clinical et al. were identified. Searches were conducted in the PubMed, Web of Science, Google Scholar, and IEEE Xplore databases using the Boolean operators “AND” and “OR” to combine keywords. Search data were recorded throughout the process. A review of the initially retrieved articles involved summarizing the index titles and keywords, conducting a secondary collection of free terms in each database, and organizing free terms. The search scope was expanded to obtain more precise or comprehensive results. Three researchers conducted a one-week discussion in July 2023 to finalize the research topic and retrieval strategy. Two trained researchers screened the relevance of article titles and abstracts to the research topic, with cross-checking by another reviewer. The first and corresponding authors performed final full-text reviews of included articles and submitted the results for collective team discussion. The literature search results are shown in Table 1.

**Table 1:** Retrieval strategy

Database	Retrieval type
PubMed	(i) “psychology” OR “psychic” OR “psychological” OR “mental” OR “mind” OR “mood” OR “sentiments” OR “emotion” OR “emotional” OR “feel” OR “affective” OR “face” OR “facial” OR “brain wave” OR “speech” OR “voice” OR “electroencephalogram” OR “EEG” OR “electrocardiogram” OR “ECG” (ii) “identify” OR “identification” OR “recognition” OR “monitor” OR “survey” OR “express” OR “perception” OR “process” OR “track” OR “recognize” (iii) “clinical” OR “patient” OR “medical treatment” OR “health care” *(i) AND (ii) AND (iii) * Results by year: 2013–2023
Google Scholar	
IEEE Xplore	
WOS	

### Eligibility criteria

#### Inclusion criteria

- Papers published in English only.
- Research published in 2013–2023.
- Studies in which the participants were patients with mental or physical disorders or eligible patient populations were extracted from publicly available databases.
- Articles that proposed or developed at least one method, model, procedure or system for emotion monitoring.

#### Exclusion criteria

- Duplicate articles were retrieved from different databases.
- Abstracts, conference minutes and reports that could not be obtained by searching or contacting the authors.
- Abstracts and original articles that were not related to the topic of the study.
- Studies that focused on emotions exhibited by patients in response to external stimuli rather than emotions identified using a certain method or technology.

**Fig. 1:**
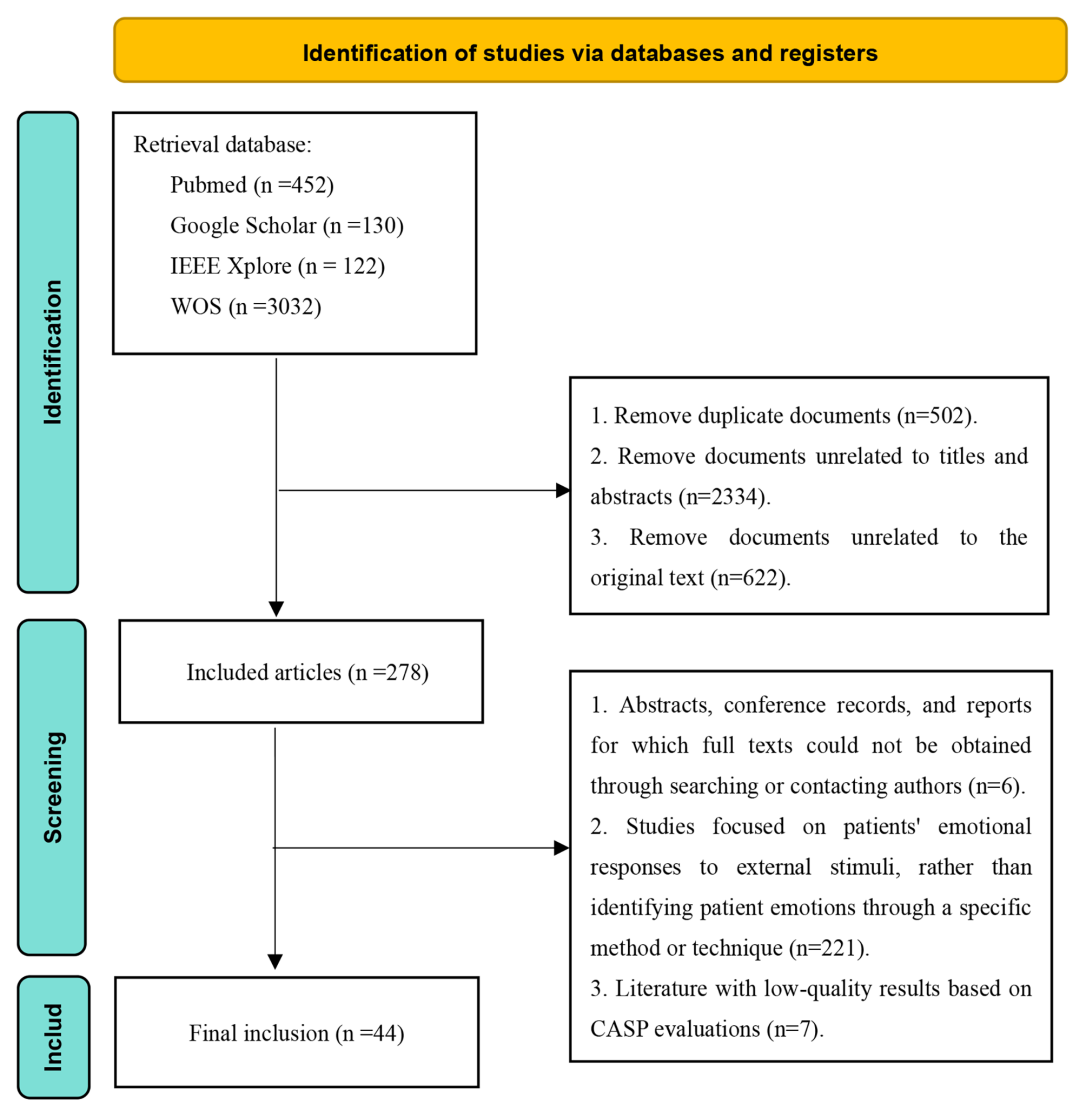
Flow chart of research screening

A manual search was conducted across four databases (see Fig. 1). A total of 3736 articles were identified, and their titles and abstracts were transferred to the reference management software EndNote 20. After duplicates were removed (*n* = 502), 3234 unique studies were identified and screened using the inclusion/exclusion criteria. The majority (*n* = 2334) of studies were excluded at the title and abstract screening stage, with an additional 622 excluded during full-text screening. The documents excluded for other reasons included abstracts for which the full text could not be obtained through a search or by contacting authors, conference proceedings, or reports (*n* = 6); studies focusing on patients’ emotional responses to external stimuli (*n* = 221); and low-quality outcome literature based on the CASP assessment (*n* = 7). Finally, 44 articles were selected for review.

### Article quality evaluation tool

The Critical Appraisal Skills Programme (CASP) criteria were adapted from the 1994 version of the “Users’ Guides to the Medical Literature” published by the American Medical Association. In this study, the CASP criteria were employed to assess the quality of the studies [21]. The CASP criteria comprise 10 items, each with three response options: “yes,” “no,” and “unclear.” The greater the number of “Yes” responses is, the greater the quality of the literature. Based on the assessment outcomes, the included literature was categorized into three levels according to quality: high, moderate, and low. To ensure the quality of the systematic review, studies with low appraisal results (i.e., with more than 3 “no” and “unclear” responses) were excluded. The detailed CASP evaluation results can be found in Appendix 1.

### Data extraction

Data were extracted independently by two researchers who were trained in data extraction, and the data were cross-checked by another researcher. Relevant data were manually extracted, including the first author’s name, year of publication, country of publication, title, DOI number, type of research, research method, purpose of the emotion recognition method, emotion recognition technology, data collection device, sample set, application scenarios, modeling foundation, psychological/emotional categories, statistical analysis method, and results.

The bias risks and types assessed in the individual studies included those proposed by the Cochrane Collaboration, such as selection bias, performance bias, detection bias, attrition bias, reporting bias, and other biases [22]. Any discrepancies or uncertainties related to bias assessment were resolved through discussions between the authors and relevant experts.

## Results

Among the 44 selected articles, 24 were experimental studies, 18 were observational studies, and 2 were mixed-methods studies. The data from 10 articles were sourced from public datasets, while the data from 33 articles were obtained through institutional recruitment. The patient populations discussed in these articles included individuals with mental disorders (BD, autism spectrum disorder, depression), neurological conditions (stroke, epilepsy, facial paralysis, facial numbness), cancer, and genetic alopecia.

**Fig. 2:**
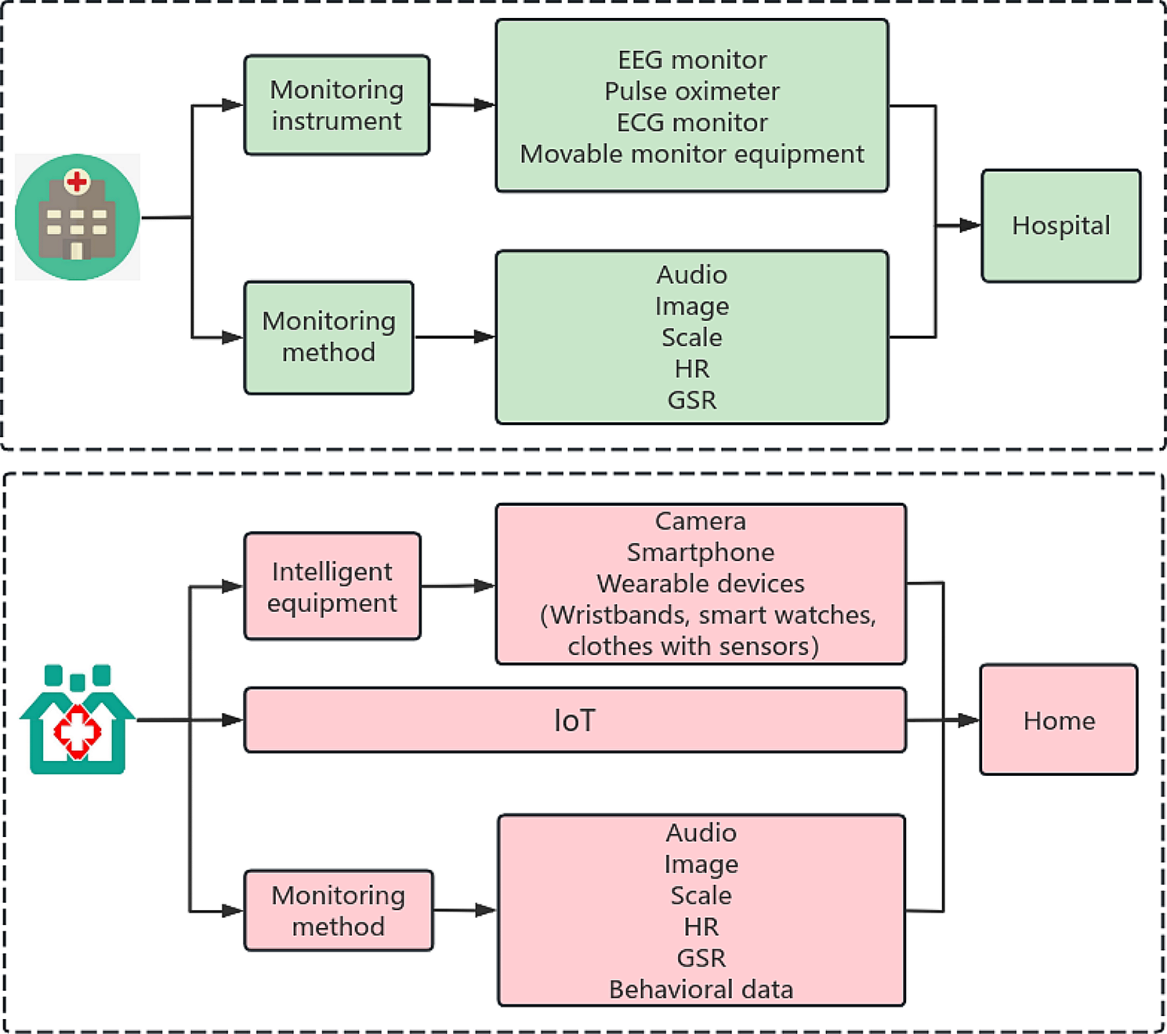
Overview of the application of emotion recognition methods

The primary application scenarios addressed in the selected articles were hospital treatment and home healthcare. Emotional recognition methods predominantly involve the utilization of scales, speech analysis, facial features, physiological signals, or multimodal techniques to construct models and systems. Research has indicated that through clinical validation (diagnosis, intervention, and treatment), certain emotion monitoring devices demonstrated good performance in reducing morbidity and mortality rates and enhancing quality of life [23, 24]. For a detailed overview, please refer to Fig. 2.

### Application of emotion recognition methods based on different scenes

Twenty-seven studies focused on hospital applications, 11 studies were conducted in outpatient or home monitoring settings, and the remaining 6 studies indicated applicability across all scenarios.

#### Application of emotion recognition technology in hospitals

In previous medical practices, most doctors or experts diagnosed patients’ emotional issues by using invasive devices and medical assessments. Automatic emotion recognition methods assist doctors not only in evaluating the overall condition of patients but also in accurately identifying diseases associated with emotional features in real time. In some studies, clinical disease features and emotional characteristics were combined as unique biomarkers that are involved in the clinical diagnostic process. They have also been used to assess patients’ performance during treatment and to aid in implementing psychological intervention therapies [25, 26].

In a study involving psychiatric patients, Masulli (2022) introduced a data-driven eye-tracking model [25]. The focus of this study was on the use of a cross-diagnostic approach to link clinical dimensional scores with eye gaze behavior. A study by Quirien (2022) suggested that the regular use of the European Organization for Research and Treatment of Cancer Core Quality of Life questionnaire (EORTC QLQ-C30) emotional function (EF) scale for screening anxiety and depression symptoms in glioma patients contributes to the early identification of emotional disorders. This practice serves as a foundation for referrals and treatment decisions [26]. Overall, these studies indicate that psychological care and interventions can enhance patients’ mental well-being within a clinical practice setting. Hence, achieving accurate and efficient emotion recognition and continuous monitoring is the initial step toward improving patients’ conditions.

#### Application of emotion recognition technology in home environments

In recent years, the development of the IoT has driven rapid advancements in the field of healthcare, leading clinical practitioners to focus on home-centered care models. Medical devices connected through the IoT offer users the opportunity to receive in-home treatment and rehabilitation, thereby alleviating pressure on healthcare systems. The application of emotional recognition methods in the “home health” domain has sparked significant interest among researchers. Faccio (2018) developed an electronic health tool based on the Family Resilience (FaRe) questionnaire aimed at monitoring the emotional state of cancer patients while at home [27]. This tool not only provides diagnostic criteria for physicians but also allows for the formulation of corresponding intervention measures. Veerbeek (2013) created a web-based psychological monitoring data collection system called “Monitoring the Mental Health of the Elderly” [28].

These systems exhibit the novelty of coordinated hardware and software. Participants complete questionnaires or scales on smart devices at scheduled times each day while their sleep and behavioral activity are continuously monitored through devices such as pulse oximeters, cameras (smart mobile devices, home surveillance devices, computers, etc.), and wearable devices equipped with sensors (e.g., tracking phone and text message usage, social interactions, and generated movement data). All these data are stored on cloud servers and fed to the backend in real time, facilitating easy access to medical information and monitoring services and ultimately reducing diagnosis time. Overall, the research indicates that the intelligent emotion recognition systems used in home environments must possess smart terminals and home treatment platforms.

### Emotion recognition technology based on different patterns

#### Emotion recognition based on psychometric scales

Fifteen articles assessed the effectiveness of the scales for emotion recognition, as shown in Table 2.

**Table 2:** Overview of emotion recognition based on psychometric scale

No	Author & year	Scale Types	N	Emotions	Test sample	Application	Statistical methods
1	A. Tsanas (2016)	MZ self-made questionnaire	130	Anxious, Elated, Sad, Angry, Irritable, Energetic	^1^BD, ^2^BPD patients	AMoSS Android application	^3^PCA
2	Quirien Oort (2022)	QLQ-C30, QLQ-BN20, HADS	99	Strain, Anxiety, Depression, Irritable	Grade II-IV gliomas patients	^4^NA	Spearman correlation, Mann-Whitney U test
3	Silvia Clausi (2014)	SCL-90-R, POMS, HDRS	70	Depression	Cerebellar ataxia patients	Mood monitoring equipment(MoMo)	Pearson, T-test
4	Vazquez Montes(2018)	ASRM, QIDS	146	Mania, Depression	BD patients	True colours self-management system	Primary analysis, Secondary analysis
5	Faccio Flaviao(2018)	FaRe questionnaire	120	Communication and cohesion, Perceived family coping, Religiousness and spirituality, Perceived social support	Cancer patients	Personal health record system (named iPHR)	NA
6	Emma Incecik(2020)	QIDS-SR, WSAS	21	Depression	Depression patients	True colours self-management system	Topic analysis
7	McIntyre S. (2023)	Mood disorders questionnaire	100	Mania, Depression	Patients of In a U.S. health plan	Rapid mood screener(RMS)	Unidirectional sensitivity analysis
8	Andrés Cárcamo(2021)	PHQ-9	6	Depression	Depression patients	MonDep APP	NA
9	Veerbeek M (2013)	The dutch version of the national health outcomes scale 65+	836	Anxiety, Depression	Older adults enrolled in outpatient mental health care	Mental health care monitor older adults (MEMO)	Descriptive statistical analysis
10	Barros Jorge (2020)	OQ-45.2, STAXI-2, RFL, DEQ, APGAR	650	Depression, Suicidal tendency, Vulnerability	Mental health patients with symptoms of emotional anxiety	NA	R statistics
11	Melbye, Sigurd Arne(2021)	MDI, ASRM, IPAQ, PSQI	206	Mania, Depression	BD patients, Unaffected relatives	Monsenso self-monitoring system	Linear mixed effects model, Chi-square test
12	Rocamora Rodrigo(2021)	HADS, STAI, BDI-II	493	Anxiety, Depression	Epilepsy patients	NA	Normality test, Chi-square test, Fisher’s exact statistics, T-test, Mann-whitney/ Kruskal-wallis test, Bonferroni
13	Dubad Muna (2021)	Self-assessment, DERS-SF	47	Positive and negative emotions	Mental health problems	“Catch It”APP	ANOVA
14	Cruz, Breno Fiuza(2022)	PANSS, CDSS, FERT-100, BACS	193	Depression	Schizophrenia patients	NA	Kolmogorov-smirnov, Pearson, Spearman, Student’s/Mann-whitney U test, χ2 test, Cronbach, Multiple linear regression analysis
15	Gangeri Laura(2022)	Cancer patient pandemic emotion questionnaire	341	NA	Cancer patients	NA	^5^EFA, ^6^CFA

^1^BD:Borderline Disorder; ^2^BPD:Borderline Personality Disorder; ^3^PCA: Principal Component Analysis; ^4^NA:Not Applicable; ^5^EFA: Exploratory Factorial Analysis; ^6^CFA: Confirmatory Factor Analyses

Emotional chart tools can assist patients in understanding their medical condition, identifying warning signs of adverse emotional episodes and relapses, and describing the instability of individual emotions. Embedding these scales in monitoring systems and applications based on momentary assessment tools can compensate for the limitations of traditional paper-based emotional chart tools due to environmental constraints. Moreover, retrospective reporting eliminates the impact of inaccurate assessment results caused by factors such as measurement outcomes, cognitive levels, and understanding errors, for example, low compliance and potential recall bias [29–31].

Tsanas (2016) [32] indicated that the Automated Monitoring of Symptom Severity (AMoSS) application system, which was embedded in smartphones and based on the mood zoom scale, enabled efficient, long-term, and effective daily emotional monitoring for patients with mood disorders. Throughout the entire process, participants exhibited good compliance, and the data were quantitatively processed and more easily preserved.

#### Emotion recognition based on speech

Three articles assessed the effectiveness of speech-based emotion recognition, as shown in Table 3.

**Table 3:** Overview of emotion recognition based on speech

No	Author & year	Feature extraction	N	Emotions	Test sample	Pattern recognition methods	Accuracy
1	Chin, Kuan-Chen (2021)	^1^MFCC	337	Stability, Unstable	Scheduling records of out-of-hospital cardiac arrest	SVM	92.87%
2	Ning JIA (2021)	Speed of speech, short-term averagy anegy, pitch frequence, MFCC	NA	Depression	AVi-D dataset	^2^GAN, ^3^CNN	67%
3	Rejaibi, Emna (2022)	Spectral, Cepstral, Glottis,Prosodic, Voice Quality, MFCC	NA	Depression	DAIC-WOZ dataset, RAVDESS dataset, AVi-D dataset	^4^DL	76.27%

^1^MFCC: Mel Frequency Cepstrum Coefficient; ^2^GAN: Generative Adversarial Network; ^3^CNN: Convolutional Neural Networks; ^4^DL: Deep Learning

Mel-frequency cepstral coefficients (MFCCs) have been widely used in speech-based emotion recognition [33–37]. Several studies have shown that support vector machine (SVM) classifiers can group multidimensional datasets by identifying hyperplanes. Chin KC (2021) used the “MFCC + SVM” approach in their research, and the results showed that the prediction accuracy, positive predictive value, negative predictive value, sensitivity, and specificity were 92.87%, 84.62%, 93.57%, 52.38%, and 98.64%, respectively [38].

Furthermore, deep convolutional neural networks (DCNNs) group multidimensional datasets by recognizing data features through recursion and iteration. Rejaibi (2022) tested the Distress Analysis Interview Corpus/Wizard-of-Oz (DAIC-WOZ) database, Ryerson Audio-Visual Database of Emotional Speech and Song (RAVDESS) dataset, and Anonymized Videos from Diverse countries (AVi-D) dataset using the “MFCC + DCNN” framework, achieving an overall accuracy of 76.27% [39]. These consistent results demonstrate that speech-based emotion recognition technology has also become an independent and viable application.

#### Emotion recognition based on facial expression

Twelve articles evaluated the effectiveness of facial expression-based emotion recognition, and the results are shown in Table 4.

**Table 4:** Overview of emotion recognition based on facial expression

No	Author & year	Feature extraction	N	Emotions	Test sample	Pattern recognition methods	Accuracy
1	Madrigal Garcia (2018)	Facial image	34	Sadness, Fear	Patients at risk of clinical deterioration	Pearson, Chi-square test, T-test, Cluster analysis, ^1^LR	NA
2	Xin Chen (2019)	Facial image	49	NA	Inpatients	CNN	Training is 92%, Test is 83%
3	Isabelle Chiu (2015)	Facial stimulation	49	Anger, Disgust, Fear, Happiness, Sadness, Astonished	Healthy individuals aged between 52 and 79	^2^GLMM	NA
4	Kowallik Andrea E (2021)	Facial action unit	55	Anger, Disgust, Fear, Happiness, Sadness, Astonished	Patients with different degrees of autism	Multinomial Logistic Regression Algorithm	F(1, 53)= 2.428, *p* = 0.125, ηp^2^ = 0.044
5	Onyema, Edeh Michael (2021)	Facial image	NA	Anger, Disgust, Fear, Happiness, Sadness, Astonished, Neutral	FER 2013 dataset	CNN	70%
6	Masulli Paolo (2022)	Eye tracking data	111	Autism, Depression, Attention deficit	Psychiatric outpatients	PCA, Linear regression	NA
7	Parra-Dominguez (2022)	Eyebrows, eyes, mouth (angle, slope, Euclidean distance, perimeter of a closed shape)	120	NA	MEEI database	5-fold cross validation, ^3^ANN	90.25%
8	Toshiya Akiyama (2022)	Facial action unit and image	71	Anger, Disgust, Fear, Happiness, Sadness, Astonished, Neutral	Schizophrenics	Multi-task CNN	66.29%
9	Jiayu Ye (2022)	Facial action unit and image	164	Anger, Disgust, Fear, Happiness, Sadness, Astonished, Neutral	Depression patients	Spearman correlation analysis, Random forest, ^4^LR-RFE	41.7%
10	Muhammad Munsif (2022)	Facial image	70	Normal, Happy, Angry, sad	KEDF dataset	CNN	Training is 96%, Test is 97%
11	Kuttenreich, Anna-Maria (2022)	Facial image	60	Anger, Disgust, Fear, Happiness, Sadness, Astonished	Patients with facial linkage after paralysis	NA	FER accuracy 67.7 ± 11.3%, AER accuracy 67.7 ± 11.3%
12	Yiming Fan (2022)	Facial image	84	Anger, Pain, Tension, Happiness, Sadness, Astonished, Fatigue, Neutral	RAF-DB dataset, FER + dataset, Private dataset of stroke patients	CNN	FER+:88.21%, RAF-DB:89.44%, private datasets 99.81%

^1^LR: Logistic Regression; ^2^GLMM: Generalized Linear Mixed Model; ^3^ANN: Artificial Neural Network; ^4^LR-RFE: Recursive Feature Elimination based on Logistic Regression

Facial expression-based emotion recognition technology utilizes computer vision and artificial intelligence to identify a person’s psychological emotions [40]. Rapid and subtle microexpressions are among the most useful external indicators for detecting hidden emotional changes. Ekman annotated static and dynamic expression in microexpression videos within the Facial Action Coding System (FACS) [41] (related datasets include the Facial Expression Recognition 2013 (FER 2013) dataset [42] and the Real-world Affective Faces Database (RAF-DB) [43]).

Convolutional neural networks (CNNs) have the ability to rapidly capture changes in facial position and image scale, and they have made significant advancements in pattern recognition, particularly in tasks such as facial detection [44] and text recognition [45]. The visual transformer (ViT) is a powerful artificial intelligence technology capable of recognizing or classifying objects within images [46, 47]. As the algorithmic performance of ViT has continued to improve and advance, it has gradually outperformed CNNs on small- and medium-sized image classification datasets [41, 48]. Jiayu Ye (2022) proposed a depression vision transformer (Dep-ViT) model to address the facial expression recognition problem in patients with depression. Compared to four other excellent models (the deep-emotion, ResNet, SCN, and ViT models), the Dep-ViT model achieved the highest accuracy [49].

#### Emotion recognition based on physiological signals

Three articles assessed the effectiveness of emotion recognition based on physiological signals, and the results are shown in Table 5.

**Table 5:** Overview of emotion recognition based on physiological signals

No	Author & year	Feature extraction	N	Emotions	Test sample	Pattern recognition methods	Accuracy
1	Prima Dewi Purnamasari (2015)	Relative wavelet energy, EEG	72	^1^HAHV, ^2^LAHV, ^3^LALV, ^4^HALV	Patients	Back propagation neural network	92.03%
2	Claudio Gentili (2016)	ECG	8	Mania, Depression	BD patients	SVM Friedman test	68.57 ± 8.01%
3	Verma, Aakash (2018)	Skin conductivity, heart rate	NA	Happiness, Sadness, Anger, Neutrality	Patients	NA	NA

^1^HAHV: High Arousal High Valence; ^2^LAHV: Low Arousal High Valence; ^3^LALV: Low Arousal Low Valence; ^4^HALV: High Arousal Low Valence

Physiological signals can provide a relatively objective reflection of an individual’s emotional state, increasing the accuracy of emotion recognition systems based on physiological signals. These physiological signals include galvanic skin response (GSR) signals, electromyographic (EMG) signals, electroencephalogram (EEG) signals, heart rate, and respiration, among others. In their research, Verma Aakash (2018) developed an emotion recognition wearable system based on Arduino for individuals with behavioral disorders [50]. This system measures skin conductivity using a GSR sensor and skin transparency using a pulse sensor and provides real-time heart rate data.

Gentili’s (2016) research indicated that combining physiological parameters with behavioral data allows for more accurate identification of subtle emotional changes [51]. Compared to changes in speech and facial expressions, the rhythmic variations in behavioral data are more representative. At present, the available physiological signal data are limited, and it is necessary to establish a complete and high-quality physiological signal database and to explore emotion models based on cognitive mechanisms combined with physiological signals.

#### Emotion recognition based on multimodality

Ten articles assessed the effectiveness of multimodal emotion recognition, and the results are shown in Table 6.

**Table 6:** Overview of emotion recognition based on multimodality

No	Author & year	Feature extraction	N	Emotions	Test sample	Pattern recognition methods	Accuracy
1	M. Shamim Hossain (2016)	Video, Audio	100	Pain, Tension, Normal	College students	GMM	99.4%
2	Amico F (2016)	^1^ERP, GSR, ^2^HRV, facial emotion and degree of pupil dilation	48	Fear, Sadness, Joy, Anger, Disgust, Surprise	Depression patients, BD patients, BPD patients	NA	NA
3	Gillian M. Sandstrom (2016)	Self-report, psychological, daily behavior	NA	Mania, Depression	BD patients	Mean, Standard deviation, Friedman test, SVM	NA
4	Xinfang Ding (2019)	EEG, eye tracking information, GSR	348	Depression	^3^MDD patients	Chi-square test, T test, Random forest, LR, SVM	Accuracy is 79.63% Precision is 76.67%
5	Bai Ran (2021)	Clinician rating scale, Sself-rating scale, telephone usage data, sleep data, step data	334	Steady-remission, Steady-depressed, Swing-drastic, Swing-moderate	MDD patients	SVM, KNN, DT, Naïve bayes, RF, LR	Steady-depressed: 84.27% Swing-drastic: 85.33%
6	Geerling B (2021)	Graphical representation of mood swings, online monitoring of sleep	17	NA	BD patients	^4^LCM	NA
7	Haiyun Huang (2021)	Behavior scale, EEG, pupillary response, gaze distance	18	Happiness, Anger, Sadness	Disturbance of consciousnes	Spectral turbulence measurement, SVM-RFE	91.5 ± 6.34%
8	Mano, Leandro Y (2016)	Images, physiological signals	NA	Neutrality, Happiness, Sadness, Fear, Anger, Surprise	Extended Cohn-Kanade (CK+) dataset	T test, Wilcoxon rank sum test, ^5^KNN, ^6^DT, Fuzzy logic, Bayesian network, SVM	99.75%
9	Yuying Tong (2020)	HRSD, HAMA, EEG, facial expression	50	Depression	Depression patients	Three-factor repeated measurement variance analysis	Happy: 87.68 ± 7.50% Neutral:82.87 ± 10.14% Sad: 75.06 ± 13.32%
10	Yulong Li (2021)	SAS, SDS, HAMD, EEG	54	Depression	Androgen alopecia patients	FAW-FS algorithm, ANOVA, Mutual information, χ2 test, LR, DT, KNN, SVM, ^7^RF	LR: 80.87% DT: 79.24% KNN: 80.42% SVM: 83.07% RF: 81.45%

^1^ERP: Event-Related Potentials; ^2^HRV: Heart Rate Variability; ^3^MDD: Major Depressive Disorder;^4^LCM: Life Cycle Management; ^5^KNN: K-Nearest Neighbors; ^6^DT: Decision Trees; ^7^RF: Random Forest

In previous research, most emotion recognition technologies relied primarily on single modalities and lacked multiple-dimensional parameters. An increasing number of studies are developing more comprehensive and optimized emotion recognition systems by incorporating various forms of data, such as psychometric questionnaire, audio signal, facial expression, EEG, and electrocardiogram (ECG) data. Hossain (2016) achieved a high recognition rate of 99.4% in a patient emotion recognition system based on a Gaussian mixture model (GMM) by combining facial expressions and audio signals [52]. In Yuying Tong’s (2020) work, a method that combines EEG and facial expression features to identify the emotions of patients with depression was proposed. This research validated the effectiveness of facial expression classification for different emotions in patients with depression and showed significant accuracy through repeated measurements [53].

### Different clinical applications of emotion recognition

Emotional recognition technology has various applications in the clinical field, positively impacting clinical research and leading to precise diagnoses, interventions, and treatments, with the potential to enhance patients’ mental health and treatment outcomes. The results are summarized in Table 7.

**Table 7:** Overview of emotion recognition applications

No	First author	Application scenario	Type of disease	Sample size	Auxiliary diagnosis	Treat	Intervene
1	M. Shamim Hossain	hospital or home	NA	100	✓	×	×
2	A. Tsanas	home	BD	130	✓	×	×
3	Madrigal Garcia	hospital	Patients in severe or critical condition	34	✓	×	×
4	Quirien Oort	hospital	Glioma	99	✓	×	×
5	Xin Chen	hospital	Inpatients	49	✓	×	×
6	Amico F	hospital	Depression, BD and borderline personality disorder	48	✓	×	×
7	Silvia Clausi	hospital or home	Cerebellar ataxia	70	✓	×	×
8	Isabelle Chiu	hospital	NA	49	✓	×	×
9	Prima Dewi Purnamasari	hospital	NA	72	✓	×	×
10	Gillian M. Sandstrom	home	Outpatients who need to quit smoking	×	✓	✓	✓
11	Claudio Gentili	hospital or home	BD	8	✓	×	×
12	Vazquez Montes	home	BD	146	✓	✓	✓
13	Faccio Flavia	home	Cancer	120	✓	✓	✓
14	Ding Xinfang	hospital	MDD	348	✓	×	×
15	Emma Incecik	home	Depression	21	×	✓	×
16	Kowallik Andrea E	hospital	Autism	55	×	✓	✓
17	Bai Ran	home	MDD	334	✓	×	×
18	Dubad Muna	home	Mental health problem	47	×	✓	✓
19	Chin, Kuan-Chen	hospital	Out of hospital cardiac arrest	337	✓	×	×
20	Geerling B	home	BD	17	×	✓	✓
21	Onyema, Edeh Michael	hospital	FER2013 data set	×	✓	×	×
22	Masulli Paolo	hospital	Psychosis	111	✓	×	×
23	Parra-Dominguez	hospital	MEEI data set	120	✓	×	×
24	Gangeri Laura	hospital	Cancer	341	✓	×	×
25	Toshiya Akiyama	hospital	Schizophrenia	71	✓	×	×
26	McIntyre, Roger S.	hospital	Health care personnel	100	✓	×	×
27	Huang, Haiyun	hospital	Mental illness disturbance of Consciousness	18	✓	×	×
28	Andrés Cárcamo	hospital	Depression	6	×	✓	×
29	J. Ye	hospital	Depression	164	✓	×	×
30	Ning JIA	hospital	Depression	×	✓	×	×
31	Mano, Leandro Y	home	Extended Cohn-Kanade data set	×	✓	×	×
32	Rejaibi, Emna	hospital	DAIC-WOZ corpus RAVDESS data set AVi-D data set	×	✓	×	×
33	Verma, Aakash	hospital or home	Behavior disorder	×	✓	×	×
34	Ye, Jiayu	hospital or home	Depression	164	✓	×	×
35	Munsif, Muhammad	hospital	KEDF public data set	70	✓	✓	×
36	Veerbeek M	home	Mental problems (depression disorder, adjustment disorder, anxiety disorder)	836	×	✓	×
37	Barros, Jorge	home	Anxiety disorders	650	×	×	✓
38	Melbye, Sigurd Arne	hospital	BD	206	×	✓	×
39	Tong, Yuying	hospital	Depression	50	✓	×	×
40	Rocamora, Rodrigo	hospital	Epilepsy	493	✓	×	×
41	Li, Yulong	hospital	Androgen alopecia	54	✓	×	×
42	Kuttenreich, Anna-Maria	hospital	Facial paralysis	60	✓	×	×
43	Fan, Yiming	hospital	RAF-DB data set FER + data set Private data set of stroke patients	84	✓	×	×
44	Cruz, Breno Fiuza	hospital or home	Schizophrenia	193	✓	×	×

Modern healthcare and nursing prioritize not only fundamental medical treatment but also psychological therapy. Clinical practitioners and healthcare professionals utilize extensive emotional monitoring data to facilitate their understanding of clinical outcomes. Research indicates that rapid psychological diagnostic results can be obtained through smart applications and instantaneous assessment techniques [54, 55]. This not only addresses the challenges faced by patients who must travel long distances for medical consultations but also streamlines the medical consultation process.

Clinical doctors can use emotional monitoring data to formulate coping strategies and relapse prevention plans. Research has shown that through self-monitoring and labeling emotional behaviors, patients can gain a better understanding of their emotions and take measures to prevent more severe emotional issues, thereby improving their mental health outcomes [56].

## Discussion

In the past decade, the field of intelligent emotion recognition has attracted the interest of numerous researchers, leading to the development of various methods based on single or multimodal approaches to effectively identify patients’ emotional states. The recognition of patients’ emotions plays a crucial role in healthcare, including in psychological counseling [57], anxiety and stress assessments [28], and pain assessments [52].

### Evaluation of the application of emotion recognition methods

A comprehensive intelligent healthcare system enables patients to receive real-time condition monitoring, timely diagnosis and effective treatment. Through intelligent devices based on cloud computing and the IoT, patients’ emotions can be rapidly and accurately identified, with notifications sent to healthcare professionals to ensure patient safety. In addition, cloud data centers can provide data storage services, data analysis, and audiovisual data processing. These patients offer secure access to healthcare professionals when they need to evaluate patients’ emotional states [58]. Emotion recognition has evolved from initially targeting patients with mental disorders (such as depression and BD) to encompassing patients with neurological conditions (such as cerebrovascular diseases, peripheral neuropathies, and spinal cord lesions). The most extensively studied applications of emotion recognition in these patients are among patients with conditions such as epilepsy, stroke, facial paralysis, facial numbness, and coma. The common feature of such patients is that they cannot express real emotions through objective external features (such as language and facial expressions) and autonomous behaviors. Therefore, it is necessary to design an automated system to effectively detect the emotions of such patients.

Methods based on neural networks and facial features have shown good performance in recognizing the emotions of patients with facial paralysis and are highly valuable in the medical field [59]. Furthermore, it is important for healthcare professionals to consider disease severity, as the extent of organ damage can affect the ability to recognize emotions and feelings. Researchers strive to ensure that any recognition system can identify these behaviors effectively. In addition to identifying basic emotions (anger, disgust, fear, happiness, sadness, and surprise), it is important to consider the intensity of these emotions. This understanding can help healthcare professionals anticipate patients’ concerns and stress levels, facilitating appropriate treatment. EEG research has indicated that patients with depression exhibit hemispheric asymmetry in brain signals, and their EEGs show regular variations [53]. Continuous emotional monitoring can provide insights into the patterns of emotional fluctuations in patients, and comprehensive psychological interventions may be beneficial for the recovery of patients with depression. Emotional recognition systems based on cloud computing and the IoT can, to some extent, address the following four major healthcare issues for patients with emotional disturbances: the shortage of healthcare professionals, long outpatient waiting times, the inability to detect changes in patient emotions early, and the increase in additional treatment costs. Consequently, these systems can support higher-quality healthcare services, thereby enhancing patient care and treatment experiences.

### Evaluation of emotion recognition technology

#### Positive and negative effects of emotion recognition technology

The use of emotion recognition technology in healthcare offers numerous advantages. First, this technology provides a quick and convenient method for conducting emotional tests through smart devices, eliminating the delays associated with traditional paper questionnaires and increasing user compliance. Second, it enables continuous monitoring of emotional states, aiding in disease understanding and the identification of factors affecting emotions and early warnings of disease progression or relapse, thereby enhancing patient treatment and quality of life. Additionally, patients can provide timely feedback without treatment interruption, helping healthcare professionals gain a timelier understanding of their conditions and offer necessary support. Furthermore, this technology automates data storage and processing, making it easier for healthcare professionals to access and analyze patient emotion information, thereby enhancing treatment personalization. Finally, incorporating multimedia elements into emotion tests improves user engagement, ultimately enhancing the user experience and increasing participation and compliance rates.

However, there are notable concerns associated with the use of emotion recognition technology in healthcare. First, long-term emotional monitoring can put pressure on patients, especially when they are required to complete daily emotional questionnaires at specific times, potentially affecting their participation and willingness to cooperate. Second, privacy concerns loom large as patients worry that the technology could compromise their personal privacy, particularly when it relates to emotional and mental health issues, leading some patients to adopt a cautious approach and withhold information regarding their true emotional states. Additionally, an excessive range of features and options in emotion recognition applications may overwhelm patients, diverting their attention and hindering their ability to focus on the primary goal of emotional monitoring, thereby diminishing the effectiveness of these applications. These concerns necessitate careful consideration of patient well-being and privacy in the implementation of this technology.

#### Limitations of emotion recognition technology

The implementation of emotion recognition technology in healthcare involves several challenges. First, data access is a critical issue, as the early stages of technology development demand a substantial amount of data for training predictive and decision models. While public databases are widely used by researchers, common problems with research datasets, such as data imbalance and limited dataset size, can lead to disparities between the data used for training and experimentation. Second, cost is a significant consideration. While medical technology aims to reduce costs, the mining, storage, and analysis of data, along with human resource and hardware utilization, can be financially burdensome. Third, cultural differences pose challenges. Older individuals may lack access to or be unwilling to use smart devices, and participants’ engagement with online monitoring systems may vary in terms of time and extent. Additionally, differences in education levels may impact the quality of the data generated and necessitate validation efforts. Finally, there is a notable lack of consensus on security, ethics, and privacy concerns in this context, further complicating the implementation of emotion recognition technology in healthcare. Addressing these challenges is essential for harnessing the full potential of this technology while ensuring patient privacy, data quality, and cost-effectiveness.

### Future development direction

In healthcare systems and health services, automatic emotion recognition technology is already being used to monitor the conditions of patients with mental health disorders. However, the future development of this technology will not only focus on psychological conditions such as depression and anxiety but also expand to monitor the severity of diseases and conditions such as cognitive impairment.

To advance emotion recognition technology, we need to overcome the limitations of currently available methods, which primarily involve the combination of questionnaires, speech analysis, facial expressions, and physiological signals. Instead, we should consider integrating a broader range of modalities to achieve more precise emotion recognition. This innovation might include incorporating data from other sensory inputs, such as touch and taste, as well as textual and image data from social media. Furthermore, as artificial intelligence and machine learning continue to advance, emotion recognition technology should move toward automation and real-time capabilities. This shift will aid in providing more personalized and immediate healthcare services, assisting patients in better managing their emotional well-being. Finally, issues related to security, ethics, and privacy remain areas that require further research and attention. It is essential to ensure that the development of emotion recognition technology complies with ethical and legal requirements while safeguarding patient privacy and data security.

### Limitations

The limitations of this study mainly lie in the review process and the assessment criteria. During the review process, our study scope may have been constrained by the limitations of the search strategy used during the literature retrieval. Although we made efforts to cover as wide a range of literature as possible, there may still be cases where some relevant studies were overlooked. We were limited to four databases and manually searched English-language literature published in the past decade to observe and evaluate the latest international research results on this topic. However, we cannot determine whether research conducted before this time frame or in other languages or databases might contain more recent research findings. During the evaluation process, it was noted that some of the selected studies lacked sufficient detail or robustness in terms of system performance. We acknowledge that such studies may lack of representative significance. From the perspective of reviewers, the attractiveness of research methods and the novelty of performance sometimes take precedence. Although the extensive heterogeneity of the results prevented us from conducting a meta-analysis, we were able to synthesize data from many studies using a comprehensive approach with robust analytical processes, encompassing a range of different study designs. Furthermore, the included studies were assessed by the reviewers as moderate to high quality, which strengthens the conclusions that can be drawn from the synthesized results. In summary, we took measures to ensure that our search strategy was as robust as possible.

## Conclusion

This study elaborated on the potential role of emotions in disease diagnosis and treatment. Emotional recognition technology based on intelligent devices and models can support the design and implementation of emotion recognition and intervention measures. By collecting patients’ physiological signals through intelligent devices and conducting real-time analysis with emotion recognition models, healthcare professionals can better understand patients’ psychological states, guiding the formulation of diagnosis and treatment plans. Real-time monitoring of patient emotions can also serve as an indicator for assessing treatment efficacy, providing a reference for optimizing and adjusting treatment plans and thereby improving patient satisfaction and recovery rates. Most studies were conducted when patients were in a static state and had sufficient time for testing. In dynamic or complex environments, continuous emotion recognition technology for addressing emotional changes still requires further research and improvement. This includes but is not limited to the following aspects. First, it is necessary to improve the robustness of emotion recognition models so that they can effectively recognize emotions in complex environments, such as noise interference and motion interference. Second, it is necessary to further explore and develop emotion recognition methods based on multimodal data that combine multiple information sources, such as physiological signals, speech, and body movements, to improve the accuracy and reliability of emotion recognition. This is an important area for future development.

### Electronic supplementary material

Below is the link to the electronic supplementary material.

Supplementary Material 1

Supplementary Material 2

Supplementary Material 3

## Data Availability

No datasets were generated or analysed during the current study.
