# Supplementary material for: Development and application of emotion recognition technology — a systematic literature review
Source: BMC Psychol. 2024 Feb 24;12:95. doi: 10.1186/s40359-024-01581-4 (PMC10894494; doi:10.1186/s40359-024-01581-4)
Supplement: Supplementary file 1 — Supplementary Material 1 [file 40359_2024_1581_MOESM1_ESM.docx]

**Appendix 1：**CASP Evaluation Form

| **NO.** | **Author** | **Title** | **Q1** | **Q2** | **Q3** | **Q4** | **Q5** | **Q6** | **Q7** | **Q8** | **Q9** | **Q10** | **Reasults** |
| --- | --- | --- | --- | --- | --- | --- | --- | --- | --- | --- | --- | --- | --- |
| 1 | M. Shamim Hossain | Patient State Recognition System for Healthcare Using Speech and Facial Expressions | Y | Y | Y | Y | U | Y | Y | Y | Y | Y | H |
| 2 | A. Tsanas | Daily longitudinal self-monitoring of mood variability in bipolar disorder and borderline personality disorder | Y | Y | Y | Y | Y | Y | Y | Y | Y | Y | H |
| 3 | Madrigal Garcia | What Faces Reveal: A Novel Method to Identify Patients at Risk of Deterioration Using Facial Expressions | Y | Y | Y | Y | Y | Y | Y | Y | Y | Y | H |
| 4 | Quirien Oort | Is the EORTC QLQ-C30 emotional functioning scale appropriate as an initial screening measure to identify brain tumour patients who may possibly have a mood disorder? | Y | Y | Y | Y | Y | Y | Y | Y | Y | Y | H |
| 5 | Xin Chen | Real-time Patient Facial Expression Recognition Using Convolutional Neural Network | Y | Y | Y | Y | U | Y | Y | Y | Y | Y | H |
| 6 | Amico F | Multimodal validation of facial expression detection software for real-time monitoring of affect in patients with suicidal intent | Y | Y | Y | Y | U | Y | Y | Y | Y | Y | H |
| 7 | Silvia Clausi | Monitoring mood states in everyday life: A new device for patients with cerebellar ataxia | Y | Y | Y | Y | Y | Y | Y | Y | Y | Y | H |
| 8 | Isabelle Chiu | "Now I see it, now I don't": Determining Threshold Levels of Facial Emotion Recognition for Use in Patient Populations | Y | Y | Y | Y | U | Y | Y | Y | Y | Y | H |
| 9 | Prima Dewi Purnamasari | EEG Based Patient Emotion Monitoring using Relative Wavelet Energy Feature and Back Propagation Neural Network | Y | Y | Y | Y | U | Y | Y | Y | Y | U | M |
| 10 | Gillian M. Sandstrom | Opportunities for Smartphones in Clinical Care: The Future of Mobile Mood Monitoring | Y | Y | Y | Y | U | Y | Y | Y | Y | Y | H |
| 11 | Claudio Gentili | Longitudinal monitoring of heartbeat dynamics predicts mood changes in bipolar patients: A pilot study | Y | Y | Y | Y | Y | Y | Y | Y | Y | Y | H |
| 12 | Vazquez Montes | Control charts for monitoring mood stability as a predictor of severe episodes in patients with bipolar disorder | Y | Y | Y | Y | Y | Y | Y | Y | Y | Y | H |
| 13 | Faccio Flavia | Development of an eHealth tool for cancer patients: monitoring psychoemotional aspects with the Family Resilience (FaRe) Questionnaire | Y | Y | Y | Y | U | Y | U | Y | Y | Y | M |
| 14 | Xinfang  Ding | Classifying major depression patients and healthy controls using EEG, eye tracking and galvanic skin response data | Y | Y | Y | Y | Y | Y | Y | Y | Y | Y | H |
| 15 | Emma Incecik | Online mood monitoring in treatment-resistant depression: qualitative study of patients' perspectives in the NHS | Y | Y | Y | Y | Y | Y | Y | Y | Y | Y | H |
| 16 | Kowallik Andrea E | Facial Imitation Improves Emotion Recognition in Adults with Different Levels of Sub-Clinical Autistic Traits | Y | Y | Y | Y | Y | Y | Y | Y | Y | Y | H |
| 17 | Bai Ran | Tracking and Monitoring Mood Stability of Patients With Major Depressive Disorder by Machine Learning Models Using Passive Digital Data: Prospective Naturalistic Multicenter Study | Y | Y | Y | Y | Y | Y | Y | Y | Y | Y | H |
| 18 | Dubad Muna | The Clinical Impacts of Mobile Mood-Monitoring in Young People With Mental Health Problems: The MeMO Study | Y | Y | Y | Y | Y | Y | N | Y | Y | N | M |
| 19 | Kuan Chen Chin | Clinical paper Early recognition of a caller's emotion in out-of-hospital cardiac arrest dispatching: An artificial intelligence approach | Y | Y | Y | Y | Y | Y | Y | Y | Y | Y | H |
| 20 | Geerling B | How to make online mood-monitoring in bipolar patients a success? A qualitative exploration of requirements | Y | Y | Y | Y | U | Y | Y | Y | Y | Y | H |
| 21 | Edeh Michael | Enhancement of Patient Facial Recognition through Deep Learning Algorithm: ConvNet | Y | N | Y | Y | U | Y | Y | Y | Y | Y | M |
| 22 | Onyema, Masulli Paolo | Data-driven analysis of gaze patterns in face perception: Methodological and clinical contributions | Y | Y | Y | Y | Y | Y | Y | Y | Y | Y | H |
| 23 | Parra-Dominguez | Towards Facial Gesture Recognition in Photographs of Patients with Facial Palsy | Y | N | Y | Y | Y | Y | Y | Y | Y | Y | H |
| 24 | Gangeri Laura | Construction process and development stages of pandemic emotions questionnaire in cancer patients (PEQ-CP) | Y | Y | Y | Y | Y | Y | Y | Y | Y | Y | H |
| 25 | Toshiya Akiyama | Comparison of Subjective Facial Emotion Recognition and Facial Emotion Recognition Based on Multi-Task Cascaded Convolutional Network Face Detection between Patients with Schizophrenia and Healthy Participants | Y | Y | Y | Y | Y | Y | N | Y | Y | N | M |
| 26 | McIntyre, Roger S. | Total healthcare cost savings through improved bipolar I disorder identification using the Rapid Mood Screener in patients diagnosed with major depressive disorder | Y | N | Y | Y | Y | Y | Y | Y | Y | Y | H |
| 27 | Haiyun  Huang | An EEG-Based Brain Computer Interface for Emotion Recognition and Its Application in Patients with Disorder of Consciousness | Y | Y | Y | Y | U | Y | Y | Y | Y | Y | H |
| 28 | Andrés Cárcamo | MonDep App: Monitoring patients with depression using sentiment analysis of therapeutic diary entries | Y | Y | Y | Y | U | Y | Y | Y | Y | Y | H |
| 29 | J. Ye | Analysis and Recognition of Voluntary Facial Expression Mimicry Based on Depressed Patients | Y | Y | Y | Y | Y | Y | Y | Y | Y | Y | H |
| 30 | Ning JIA | Emotion Recognition of Depressive Patients Based on General Speech Information | Y | N | Y | Y | U | Y | Y | Y | Y | Y | M |
| 31 | Mano, Leandro Y | Exploiting IoT technologies for enhancing Health Smart Homes through patient identification and emotion recognition | Y | N | Y | Y | Y | Y | Y | Y | Y | Y | H |
| 32 | Rejaibi, Emna | MFCC-based Recurrent Neural Network for automatic clinical depression recognition and assessment from speech | Y | N | Y | Y | U | Y | Y | Y | Y | Y | M |
| 33 | Verma, Aakash | Emotion Recognition System for Patients with Behavioral Disorders | Y | U | Y | Y | U | Y | Y | Y | Y | Y | M |
| 34 | Ye, Jiayu | Dep-ViT: Uncertainty Suppression Model Based on Facial Expression Recognition in Depression Patients | Y | Y | Y | Y | Y | Y | Y | Y | Y | Y | H |
| 35 | Munsif, Muhammad | Monitoring Neurological Disorder Patients via Deep Learning Based Facial Expressions Analysis | Y | N | Y | Y | U | Y | Y | Y | Y | Y | M |
| 36 | Veerbeek M | Mental health care Monitor Older adults (MEMO): monitoring patient characteristics and outcome in Dutch mental health services for older adults | Y | N | Y | Y | Y | Y | Y | Y | Y | Y | H |
| 37 | Barros, Jorge | Recognizing states of psychological vulnerability to suicidal behavior: a Bayesian network of artificial intelligence applied to a clinical sample | Y | N | Y | Y | Y | Y | Y | Y | Y | Y | H |
| 38 | Melbye, Sigurd Arne | Mood, activity, and sleep measured via daily smartphone-based self-monitoring in young patients with newly diagnosed bipolar disorder, their unaffected relatives and healthy control individuals | Y | Y | Y | Y | Y | Y | Y | Y | Y | Y | H |
| 39 | Tong, Yuying | Biases of Happy Faces in Face Classification Processing of Depression in Chinese Patients | Y | Y | Y | Y | Y | Y | Y | Y | Y | Y | H |
| 40 | Rocamora, Rodrigo | Mood Disturbances, Anxiety, and Impact on Quality of Life in Patients Admitted to Epilepsy Monitoring Units | Y | Y | Y | Y | Y | Y | Y | Y | Y | Y | H |
| 41 | Li, Yulong | Research on Mental Stress Recognition of Depressive Disorders in Patients With Androgenic Alopecia Based on Machine Learning and Fuzzy K-Means Clustering | Y | Y | Y | Y | Y | Y | Y | Y | Y | Y | H |
| 42 | Kuttenreich, Anna-Maria | Facial Emotion Recognition in Patients with Post-Paralytic Facial Synkinesis-A Present Competence | Y | Y | Y | Y | Y | Y | Y | Y | Y | Y | H |
| 43 | Fan, Yiming | FER-PCVT: Facial Expression Recognition with Patch-Convolutional Vision Transformer for Stroke Patients | Y | N | Y | Y | U | Y | Y | Y | Y | Y | M |
| 44 | Cruz, Breno Fiuza | Validation of the Brazilian version of the Hinting Task and Facial Emotion Recognition Test (FERT-100) in patients with schizophrenia. | Y | Y | Y | Y | Y | Y | Y | Y | Y | Y | H |
| Note： 1. CASP criteria for qualitative studies: Q1. Did the article describe an important clinical problem addressed via a clearly formulated question? Q2.Was a qualitative approach appropriate? Q3. Was the sampling strategy clearly defined and justified? Q4.What methods did the researcher use for collecting data?Q5. What methods did the researcher use to analyse the data, and what quality control measures were implemented? Q6.Was the relationship between the researcher(s) and participant(s) explicit. Q7.What are the results, and do they address the research question? Q8.Are the results credible? Q9.What conclusions were drawn, and are they justified by the results? Q10.To what extent are the findings of the study transferable to other clinical settings? 2."Y"=Yes, "N"=No,"U"=Unclear. 3."H"=Hingher quality, "M"=Medium quality. | | | | | | | | | | | | | |
